# Long-term effects of neoadjuvant radiotherapy, adjuvant radiotherapy, and chemotherapy-only on survival of locally advanced non-small cell lung Cancer undergoing surgery: a propensity-matched analysis

**DOI:** 10.1186/s12885-018-4900-x

**Published:** 2018-11-06

**Authors:** Xinyu Wang, Chang Yin, Shaofei Su, Xi Li, Chao Wang, Chaoli Zhang, Meina Liu

**Affiliations:** 1 Department of Biostatistics, Public Health College, Harbin Medical University, 157 Baojian Road, Harbin, Heilongjiang Province 150081 People’s Republic of China; 2 Information Centre, National Institute of Hospital Administration, Beijing, China; 3 Care Quality Control Office, Xi’an Jiaotong University Second Affiliated Hospital, Xi’an, China

**Keywords:** Non-small cell lung cancer, Neoadjuvant radiotherapy, Adjuvant radiotherapy, Survival

## Abstract

**Background:**

The optimal timing of radiotherapy (RT) with respect to surgery remains controversial for locally advanced non-small cell lung cancer (LA NSCLC) undergoing surgery and the long-term effect of neoadjuvant RT, adjuvant RT, and chemotherapy-only on survival is unknown.

**Methods:**

A retrospective study with Greedy 5 → 1 Digit propensity score matching technique was performed for locally advanced NSCLC patients identified from the Surveillance, Epidemiology, and End Results (SEER) database during 2004 to 2012. Kaplan-Meier and the log-rank test were conducted to compare NSCLC-specific survival. Cox proportional hazards multivariable regression was performed to assess the impact of different treatment regimens on cancer-specific mortality after adjustment for demographic factors, histology type, tumor grade, tumor size, nodal stage, and extent of resection.

**Results:**

One thousand, two hundred and seventy-eight locally advanced NSCLC patients undergoing surgery were identified after propensity matching. Cox regression analyses showed the risk of cancer-specific mortality is not significantly different among neoadjuvant RT, adjuvant RT, and chemotherapy-only. Subgroup analyses showed that for patients with T1/2 & N2/3, the surgery plus chemotherapy-only group showed markedly higher mortality risk (HR = 1.42, 95%CI:1.10–1.83) than the neoadjuvant RT group. Other risk factors include older age, higher tumor grade, larger tumor size, and greater lymph node involvement.

**Conclusions:**

The findings of this study suggest that the benefit of additional neoadjuvant or adjuvant RT to chemotherapy may be linked to a proper selection of LA NSCLC patients who undergo surgery. The timing of radiotherapy should be decided on the premise of fully considering patients’ condition and the quality of life after treatment.

**Electronic supplementary material:**

The online version of this article (10.1186/s12885-018-4900-x) contains supplementary material, which is available to authorized users.

## Background

Lung cancer is currently the leading cause of cancer death around the world [1]. American Cancer Society has estimated that there will be 222,500 new cases and 155,870 deaths caused by lung cancer in the United States in 2017 [2], of which non-small cell lung cancer (NSCLC) constitutes about 85% [3]. Approximately one-third of NSCLC patients are diagnosed with locally advanced (LA) disease (stage IIIA/IIIB) [4]. Since this is an extremely heterogeneous group, the optimal treatment remains controversial [5–9]. Though many studies have compared the survival difference between adjuvant chemotherapy or radiotherapy (RT) and surgery alone [10–13], and between neoadjuvant therapy and surgery alone [14–17], few have investigated the difference between neoadjuvant RT and adjuvant RT, as well as the survival difference between RT plus chemotherapy and chemotherapy-only for LA NSCLC patients undergoing surgery. Although a combined modality approach of RT, chemotherapy, and surgery is routinely recommended for resectable LA patients, the optimal sequence is still under debate, and the prognosis remains poor with a high rate of distant metastasis and low five-year overall survival rate [4, 18].

The aims of neoadjuvant RT are to improve resectability through shrinking the tumor, downstage the nodal disease, sterilize micrometastases, and enhance local control by the removal of the residual tumor and nodal disease [8, 19, 20]. The disadvantages are also coexisting which show the negative impact of treatment on patients’ performance status, the technically challenging surgery after RT, and the increased rate of postoperative complications [21]. To date, no population-based evaluation of the long-term effects of neoadjuvant/adjuvant RT has been performed. Therefore, we designed a retrospective study with three propensity-score-matched groups: surgery + chemotherapy + neoadjuvant RT group (neoadjuvant RT group), surgery + chemotherapy + adjuvant RT group (adjuvant RT group), and surgery + chemotherapy-only group(chemotherapy-only group), to evaluate the effect of the sequence of RT with surgery on cancer-specific and overall survival among patients with LA NSCLC.

## Methods

### Study population and propensity score matching

**Fig. 1:**
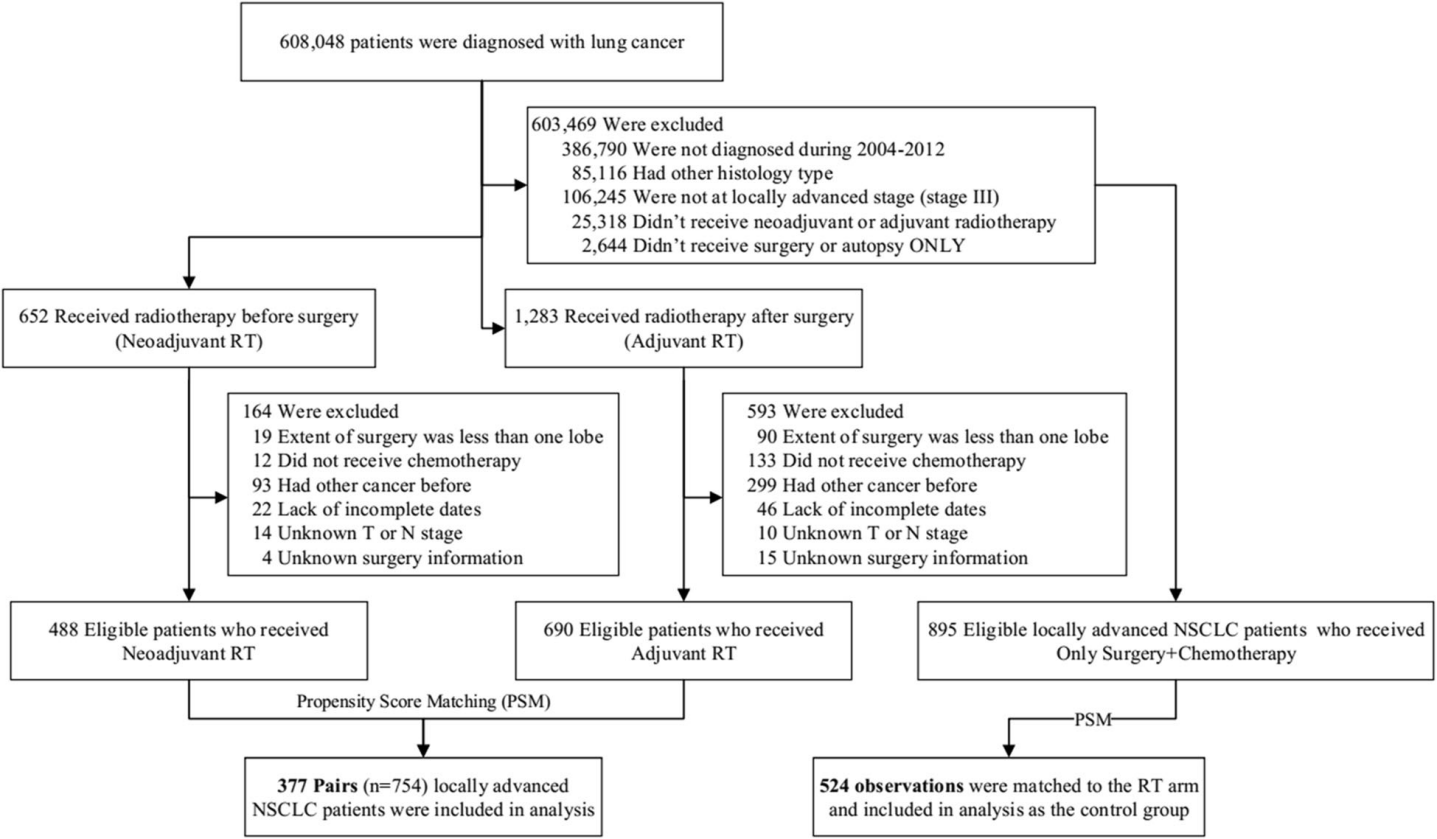
Flow chart of study patients’ enrollment

We utilized the Surveillance, Epidemiology, and End Results (SEER) database, which includes information on cancer incidence, treatment, and survival for approximately 28% of the US population, to derive a dataset of patients who were diagnosed with locally advanced NSCLC during 2004–2012, followed up until 31 December 2014. The flowchart for selecting the study sample is shown in Fig. 1. Because this dataset is within the public domain and all patient information is de-identified, it was deemed exempt from review by the Institutional Review Board, and the informed consent was waived.

Individual data for each eligible patient were retrieved from the database including age at diagnosis, gender, race, histology, tumor grade, AJCC (The American Joint Committee on Cancer) 6th T classification and N classification (all patients included in this study were at M0), radiation sequence with surgery, survival time, and vital status at last follow-up (December 31, 2014). The extent of resection was derived from SEER Site-Specific Surgery of Primary Site Codes and was grouped into two categories: “lobectomy or bilobectomy” and “pneumectomy”. The outcome of the main analyses is NSCLC-specific survival, which was calculated as the number of months from diagnosis to death due to NSCLC. Patients who died from other causes or were still alive at the end of the follow-up date were defined as censored.

To reduce selection bias and compare the effect of neoadjuvant RT and adjuvant RT, we adjusted the difference of characteristics between the two groups using propensity score matching. We used a logistic regression model with RT type as the dependent variable and other extracted variables (age at diagnosis, gender, race, histology, tumor grade, T classification, N classification, and extent of resection) as independent variables, to create the propensity score. Greedy 5 → 1 Digit Match algorithm was then applied to obtain the optimal match [22]. About 78.71% of the cases in the neoadjuvant RT group were matched to cases in adjuvant RT group, resulting in 377 pairs of patients. One hundred and-nine neoadjuvant patients did not match due to disjoint ranges of propensity scores. To increase the matched sample size of the control group (chemotherapy-only), we performed the propensity score matching procedure separately for the control group and the two RT groups. Then we merged the matched results and removed the duplicate observations, resulting in 524 patients in the chemotherapy-only group. Comparison of characteristics among the original groups is presented in Additional file 1.

### Statistical analysis

Baseline patient characteristics were compared with the one-way ANOVA or Chi-square test, as appropriate. For the outcome measure, we used the Kaplan-Meier method to evaluate NSCLC-specific survival and compared the survival curves of each group using the log-rank test. Cox proportional hazards multivariable regression was performed to assess the impact of radiation sequence with surgery on cancer-specific mortality after adjustment for demographic factors, histology type, tumor grade, tumor size, nodal stage, and extent of resection. Cox regression was also performed to identify covariates associated with increased all-cause mortality adjusting for the same variables.

Furthermore, we performed subgroup analyses, stratified by extension of resection and combinations of T classification and N classification, to examine the effect of neoadjuvant RT on cancer-specific survival for patients with different stages of the disease. For sensitivity analysis, we repeated the main analyses for the original (unmatched) dataset. All statistical analysis was performed using SAS version 9.4 software (SAS Institute, Cary, NC, USA).

## Results

### Baseline characteristics

Before propensity score matching, neoadjuvant and adjuvant RT cohorts, as well as the control group, show the significantly different distribution of potentially confounding factors including age, race, histology, tumor grade, T classification, and N classification (Additional file 1: Table S1). After propensity-score matching, all characteristics except the grade of tumor among the three groups are perfectly balanced **(**Table 1). In the matched population, the mean age was 61.03 years, male (56.26%) and predominantly white population (80.20%). 514(40.22%) and 356(27.86%) patients were diagnosed as adenocarcinoma and squamous cell lung cancer, respectively. 738(57.75%) patients were reported to have a tumor of grade III/IV. For AJCC 6th stage classification, 377(29.50%) had a tumor of T4, and 984(77.00%) patients had an N2/N3 stage.

**Table 1:** Baseline characteristics of the matched neoadjuvant and adjuvant radiotherapy cohorts

Variable	N (%)	Survival	Number of patients (%)						*P* value
		Median (IQR), mo	Adjuvant RT (*N* = 377)		Neoadjuvant RT (N = 377)		Surgery + Chemotherapy Only (*N* = 524)		
Age, years	61.03 ± 9.52	–	61.88 ± 9.86		60.23 ± 9.80		61.72 ± 9.04		0.0645
Sex									0.9669
Male	719 (56.26)	58 (18, NR)	210	(55.70)	213	(56.50)	296	(56.49)	
Female	559 (43.74)	65 (23, NR)	167	(44.30)	164	(43.50)	228	(43.51)	
Race									0.5785
White	1025 (80.20)	61 (19, NR)	303	(80.37)	309	(81.96)	413	(78.82)	
Black	152 (11.89)	79 (18, NR)	47	(12.47)	43	(11.41)	62	(11.83)	
Other	101 (7.90)	57 (26, NR)	27	(7.16)	25	(6.63)	49	(9.35)	
Histology									0.8385
Adenocarcinoma	514 (40.22)	54 (21, NR)	144	(38.20)	150	(39.79)	220	(41.98)	
Squamous cell	356 (27.86)	69 (19, NR)	109	(28.91)	104	(27.59)	143	(27.29)	
Others	408 (31.92)	79 (19, NR)	124	(32.89)	123	(32.63)	161	(30.73)	
Grade									0.0366
I/ II	389 (30.44)	66 (26, NR)	101	(26.79)	107	(28.38)	181	(34.54)	
III/ IV	738 (57.75)	48 (17, NR)	226	(59.95)	218	(57.82)	294	(56.11)	
Unknown	151 (11.82)	103 (35, NR)	50	(13.26)	52	(13.79)	49	(9.35)	
T classification									0.4202
T1	211 (16.51)	73 (28, NR)	70	(18.57)	55	(14.59)	86	(16.41)	
T2	535 (41.86)	54 (20, NR)	160	(42.44)	148	(39.26)	227	(43.32)	
T3	155 (12.13)	46 (15, NR)	40	(10.61)	54	(14.32)	61	(11.64)	
T4	377 (29.50)	66 (18, NR)	107	(28.38)	120	(31.83)	150	(28.63)	
N classification									0.2972
N0/N1	294 (23.00)	81 (21, NR)	82	(21.75)	80	(21.22)	132	(25.19)	
N2/N3	984 (77.00)	56 (19, NR)	295	(78.25)	297	(78.78)	392	(74.81)	
Extent of Resection									0.9712
Lobectomy or Bilobectomy	1056 (82.63)	64 (21, NR)	311	(82.49)	313	(83.02)	432	(82.44)	
Pneumectomy	222 (17.37)	53 (15, NR)	66	(17.51)	64	(16.98)	92	(17.56)	
Treatment regimen									–
Neoadjuvant RT	377 (29.50)	67 (20, NR)	–	–	–	–	–	–	
Adjuvant RT	377 (29.50)	61 (17, NR)	–	–	–	–	–	–	
Surgery + Chemotherapy Only	524 (41.00)	61 (22, NR)							

*NR* not reached

### Survival

**Fig. 2:**
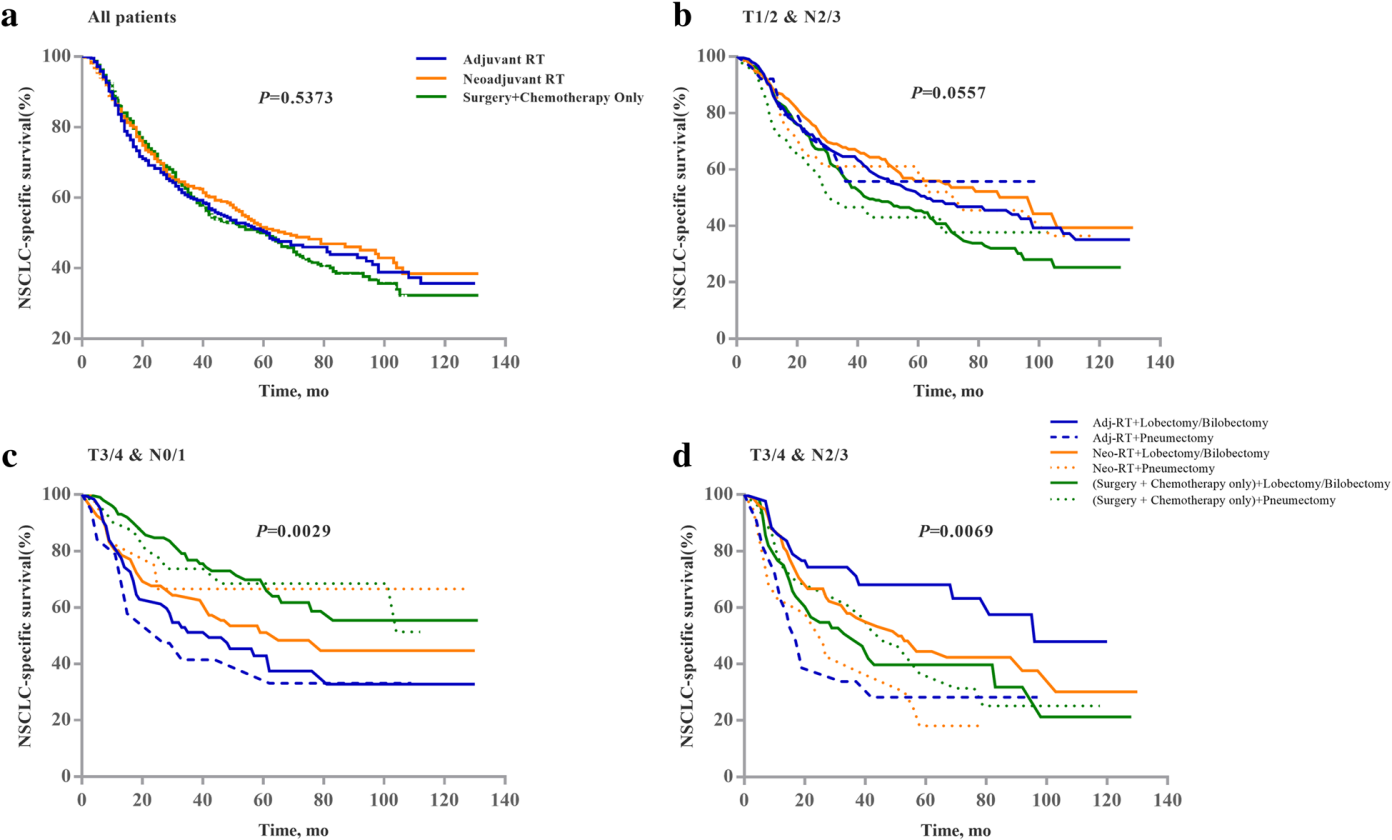
Cancer-specific survival curves compared by the Log-rank test for (**a**) all patients, (**b**) patients with T1/2 & N 2/3, (**c**) patients with T3/4 & N0/1, and (**d**) patients with T3/4 & N2/3

**Fig. 3:**
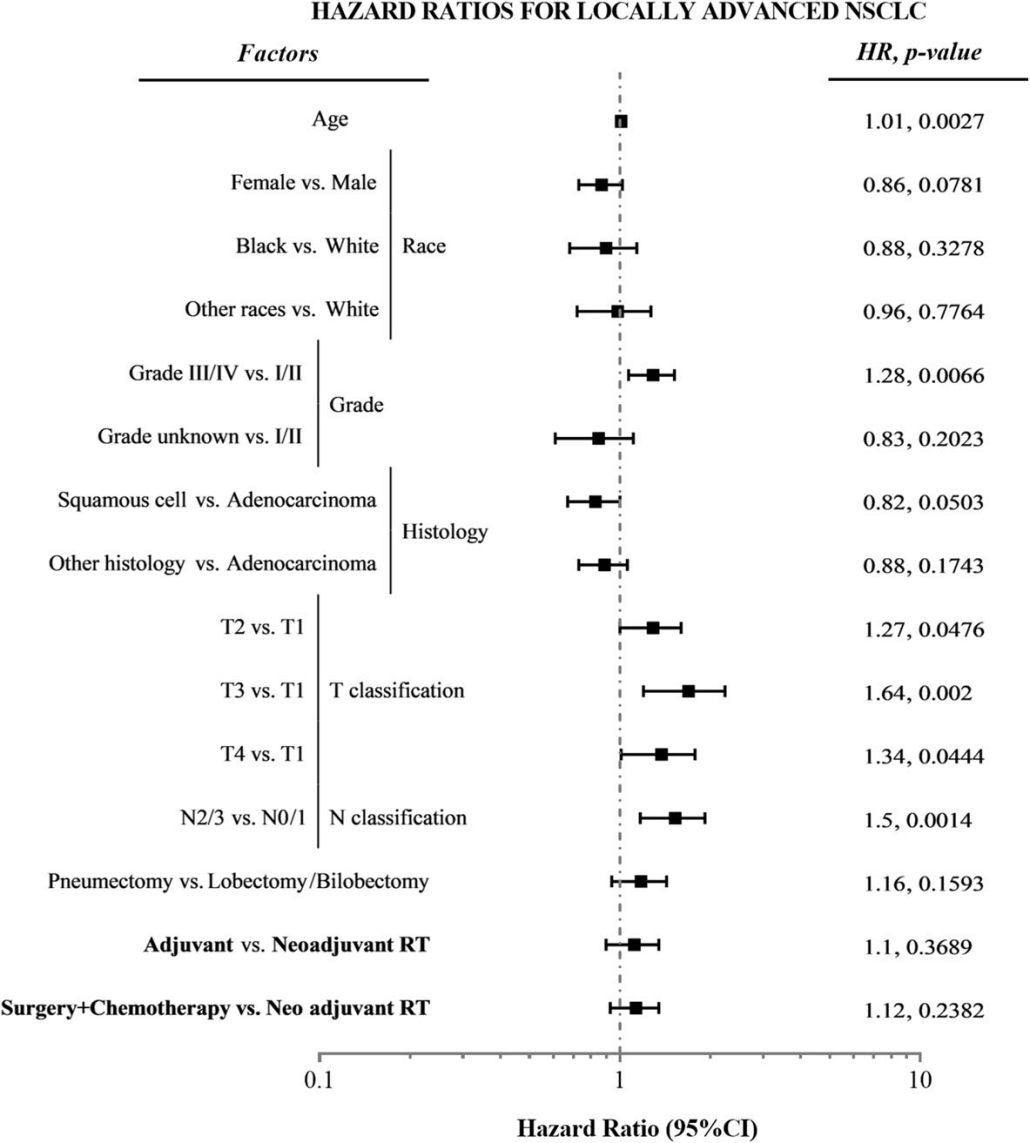
Forest plots of multivariate Cox regression analysis for cancer-specific mortality of the matched cohorts

The median NSCLC-specific survival time for neoadjuvant RT patients is 67 months, for adjuvant RT patients and the chemotherapy-only group is 61 months. The log-rank test of survival curves is statistically insignificant (*P* = 0.5373, Fig. 2a). On multivariate Cox proportional hazards regression (Table 2 and Fig. 3), the HR for cancer-specific mortality among patients receiving adjuvant RT and chemotherapy-only did not differ from that among patients receiving neoadjuvant RT (HR = 1.10, 95%CI: 0.90–1.35; HR = 1.12, 95%CI: 0.93–1.35, respectively), after adjusting key potential confounders. We observed a higher risk of cancer-specific mortality in patients with N2/N3 (vs. N0/N1; HR = 1.50, 95%CI: 1.17–1.92), higher T classification (HR = 1.27 for T2, HR = 1.64 for T3, and HR = 1.34 for T4 vs T1), tumor grade III/ IV (vs. grade I/ II; HR = 1.28, 95%CI: 1.07–1.52), older age (HR = 1.01, 95%CI: 1.00–1.02). For all-cause mortality, the results were mostly consistent with those of the analysis of NSCLC-specific mortality. We also observed a decreased all-cause mortality risk in female patients (vs. male patients; HR = 0.82, 95%CI: 0.70–0.96).

**Table 2:** Multivariable Cox regression for propensity-score matched (PSM) dataset

Variable	Cancer-Specific mortality		All-cause mortality	
	HR (95% CI)	*p-*value	HR (95% CI)	*p-*value
Age	1.01 (1.00, 1.02)	0.0027	1.02 (1.01, 1.02)	<.0001
Sex				
Male	*Ref.*		*Ref.*	
Female	0.86 (0.73, 1.02)	0.0781	0.82 (0.70, 0.96)	0.0108
Race				
White	*Ref.*		*Ref.*	
Black	0.88 (0.68, 1.14)	0.3278	0.88 (0.69, 1.11)	0.2748
Others	0.96 (0.72, 1.27)	0.7764	0.95 (0.73, 1.24)	0.7033
Grade				
I/ II	*Ref.*		*Ref.*	
III/ IV	1.28 (1.07, 1.52)	0.0066	1.26 (1.07, 1.49)	0.0052
Unknown	0.83 (0.61, 1.11)	0.2023	0.82 (0.62, 1.08)	0.1509
Histology				
Adenocarcinoma	*Ref.*		*Ref.*	
Squamous cell	0.82 (0.67, 1.00)	0.0503	0.85 (0.70, 1.03)	0.0928
Others	0.88 (0.73, 1.06)	0.1743	0.93 (0.78, 1.11)	0.4148
T classification				
T1	*Ref.*		*Ref.*	
T2	1.27 (1.00, 1.60)	0.0476	1.26 (1.01, 1.56)	0.0375
T3	1.64 (1.20, 2.24)	0.0020	1.49 (1.11, 2.00)	0.0082
T4	1.34 (1.01, 1.78)	0.0444	1.24 (0.95, 1.63)	0.1121
N classification				
N0/N1	*Ref.*		*Ref.*	
N2/N3	1.50 (1.17, 1.92)	0.0014	1.38 (1.09, 1.74)	0.0065
Extent of Surgery				
Lobectomy or Bilobectomy	*Ref.*		*Ref.*	
Pneumectomy	1.16 (0.94, 1.43)	0.1593	1.15 (0.95, 1.40)	0.1585
Treatment regimen				
Neoadjuvant RT	*Ref.*		*Ref.*	
Adjuvant RT	1.10 (0.90, 1.35)	0.3689	1.16 (0.96, 1.40)	0.1273
Surgery + Chemotherapy-Only	1.12 (0.93, 1.35)	0.2382	1.18 (0.99, 1.41)	0.0653

*HR* Hazard Ratio

Since the mortality risk was significantly different among different T and N classification, we conducted an exploratory subgroup analysis to investigate whether the patients with different combinations of the size or direct extent of primary tumor (T classification) and the degree of spread to regional lymph nodes (N classification) benefit differently from administration of neoadjuvant or adjuvant RT. We combined T1 and T2, T3 and T4, N0 and N1, N2 and N3, resulting in three subgroups: T1/2 & N2/3, T3/4 & N2/3, and T3/4 & N0/1.

We compared the cancer-specific survival curves of different combinations of the extent of resection and treatment regimen, stratified by T and N classification (Fig. 2b/c/d). The log-rank tests showed that for patients at T1/2 & N2/3, the survival curves did not differ significantly from each other (*P* = 0.0557). For patients at T3/4 & N0/1, patients receiving adjuvant RT had the lowest survival rate among the three treatment regimens, regardless of the extent of resection. Patients receiving chemotherapy only had a higher survival rate than patients receiving neoadjuvant RT when combined with lobectomy/bilobectomy. For patients at T3/4 & N2/3, a marked difference was observed between the survival curve of lobectomy/bilobectomy plus adjuvant RT and the curve of pneumectomy plus adjuvant RT. The survival rate of adjuvant RT is also higher than that of patients receiving neoadjuvant RT or chemotherapy-only when combined with lobectomy/bilobectomy.

The results of multivariable Cox regression for cancer-specific mortality for each subgroup are shown in Table 3. For patients at T1/2 & N2/3, the surgery plus chemotherapy-only group showed a significantly higher mortality risk (HR = 1.42, 95%CI:1.10–1.83) than the neoadjuvant RT group. Higher tumor grade was also a risk factor (HR = 1.34, 95%CI:1.07–1.67). For patients at T3/4 & N0/1, different treatment regimens did not show significant disparity in survival, but the tumor grade III/ IV is still a risk factor compared to grade I/ II (HR = 1.67, 95%CI:1.08–2.58). For patients at T3/4 & N2/3, patients receiving pneumectomy experienced a higher risk of cancer-specific mortality than patients undergoing lobectomy or bilobectomy (HR = 1.80, 95%CI:1.25–2.61). Older age is also a marked risk factor (HR = 1.03, 95%CI:1.01–1.05).

**Table 3:** Multivariable Cox regression for cancer-specific mortality stratified by T and N stages

Variable	T1/2 & N2/3	T3/4 & N0/1	T3/4 & N2/3
	(*N* = 746)	(*N* = 294)	(*N* = 238)
Age	1.01 (1.00, 1.02)	1.00 (0.98, 1.02)	1.03 (1.01, 1.05) **
Sex			
Male	*Ref.*	*Ref.*	*Ref.*
Female	0.81 (0.66, 1.01)	0.92 (0.63, 1.34)	0.97 (0.67, 1.40)
Race			
White	*Ref.*	*Ref.*	*Ref.*
Black	0.87 (0.62, 1.21)	0.80 (0.44, 1.45)	0.97 (0.57, 1.65)
Others	0.97 (0.68, 1.38)	1.13 (0.54, 2.34)	0.88 (0.48, 1.62)
Grade			
I/ II	*Ref.*	*Ref.*	*Ref.*
III/ IV	1.34 (1.07, 1.67) *	1.67 (1.08, 2.58) *	0.93 (0.63, 1.37)
Unknown	0.74 (0.51, 1.07)	0.66 (0.30, 1.44)	1.79 (0.93, 3.46)
Histology			
Adenocarcinoma	*Ref.*	*Ref.*	*Ref.*
Squamous cell	1.01 (0.77, 1.31)	0.69 (0.44, 1.07)	0.66 (0.42, 1.05)
Others	0.98 (0.77, 1.25)	0.94 (0.61, 1.44)	0.72 (0.47, 1.11)
Extent of Surgery			
Lobectomy or Bilobectomy	*Ref.*	*Ref.*	*Ref.*
Pneumectomy	1.04 (0.76, 1.43)	1.00 (0.63, 1.59)	1.80 (1.25, 2.61) **
Treatment regimen			
Neoadjuvant RT	*Ref.*	*Ref.*	*Ref.*
Adjuvant RT	1.11 (0.84, 1.47)	1.54 (0.99, 2.38)	0.74 (0.47, 1.16)
Chemotherapy-Only	1.42 (1.10, 1.83) **	0.65 (0.42, 1.02)	0.93 (0.62, 1.41)

* *p* < 0.05; ** *p* < 0.01

### Sensitivity analyses

We performed multivariate Cox regression for cancer-specific survival and overall survival with the pre-matched population. The results were consistent with those analyzed with the matched population that neoadjuvant RT did not show advantage or disadvantage compared with adjuvant RT or chemotherapy-only. However, we observed a slightly higher risk of cancer-specific mortality in patients receiving pneumectomy, compared with patients undergoing lobectomy or bilobectomy (see Additional file 1).

## Discussion

To our knowledge, the present study is the first to compare the effect of neoadjuvant RT, adjuvant RT, and chemotherapy-only on the long-term survival of LA NSCLC patients undergoing surgery using the large population-based SEER database and propensity score matching technique. The RT arms did not confer an additional cancer-specific or overall survival advantage beyond that achieved with surgery plus chemotherapy alone. However, for patients at T1/2 & N2/3, the chemotherapy-only group seems to be at a survival disadvantage compared with neoadjuvant or adjuvant RT groups. The risk factors for cancer-specific and overall mortality are mainly the tumor characteristics including the grade, the size, and the lymph node involvement.

A recent analysis based on the National Cancer Database [23] shows that both adjuvant and neoadjuvant chemotherapy provide superior survival outcomes compared to surgery alone, though no clear evidence is showing that neoadjuvant is superior to adjuvant in the treatment of resectable stage II and III NSCLC. In fact, whether RT can improve or not the outcomes of surgery and chemotherapy in LA NSCLC is still the subject of scientific debate, on which Robinson et al. conducted a study using large database analyses [24] and concluded that modern postoperative radiotherapy seems to confer an additional overall survival advantage beyond that achieved with adjuvant chemotherapy alone for patients with N2 NSCLC after complete resection. While a recently issued critical review by Tini et al., which discussed possible combination strategies aimed to improve the outcome of lung cancer patients, indicated the RT management of LA NSCLC is currently unsatisfactory [25]. Results from our study further confirmed that for patients with N2/3 and T1/2, all of whom received complete resection and chemotherapy, neoadjuvant and adjuvant RT confer an additional improvement in cancer-specific survival. However, such an advantage was not discovered among patients with other T and N stage.

Previous studies have shown that respectable survival can be achieved after neoadjuvant chemoradiation, followed by anatomic resection, in selected patients with clinically advanced NSCLC [26]. However, this study did not find a significant difference between neoadjuvant and adjuvant RT. There is some issue to discuss. Firstly, the patients in neoadjuvant RT group at least showed a good response, as the patients with the response evaluation of “PD” lose the opportunity to surgery, which may cause unbalance between the neoadjuvant and adjuvant RT groups. Secondly, for patients who received neoadjuvant RT, the primary tumors were harder to undergo surgery, which made the neoadjuvant RT necessary to come first and weakened the additional survival benefit of neoadjuvant RT compared to adjuvant RT. Thirdly, though not significantly different in multivariate analysis, the survival curves of neoadjuvant and adjuvant RT are much closer for patients with T1/2 & N2/3 than those of patients with T3/4 & N2/3 or T3/4 & N0/1. Besides, the survival rate of the neoadjuvant RT is a little higher than that of the adjuvant RT (combined with lobectomy/bilobectomy) for patients with T3/4 & N0/1, while the situation is reversed for patients with T3/4 & N2/3, which indicates the benefit of neoadjuvant or adjuvant RT may be linked to a proper selection of patients.

Results from our study also confirm the importance of well-established predictors of poorer outcome in patients with NSCLC, including older age and male sex [27, 28], although the absolute effect was small relative to other factors. We also demonstrated an independent effect of tumor size and higher tumor grade.

Unlike clinical trials, many factors involved in determining the course of treatment will not be captured in the registry data. Such factors include patient preferences, physician recommendations, comorbidities, and proximity to treatment providers. However, the potential selection bias originating from the retrospective design was minimized with the propensity score matching process and the subgroup analyses, and the sensitivity analyses also proved the validity and reliability of study results. Nevertheless, several limitations should be taken seriously when interpreting the results. Firstly, we chose to study the long-term effects of neoadjuvant RT, adjuvant RT, and chemotherapy-only on survival among patients who received radical surgery, but the sequence of chemotherapy with surgery and RT was unknown. Therefore, neither did we distinguish neoadjuvant or adjuvant chemotherapy in this study nor did we know whether the patient received concurrent or sequential chemoradiotherapy or the two treatment modalities were divided by surgery. Secondly, the detailed information of RT such as daily and total dose, volume of RT field, and the details of surgery such as pathologic resection margin status, lymphadenectomy, and surgical techniques of surgeons was not available thus could not be evaluated in this study. Thirdly, Other unmeasured and potential confounders included patients’ medical history and comorbidities. The simple classification of surgery according to the extent of resection may not fully reflect the details of the surgical procedures. In the future, with enough information of chemotherapy, studies on the effect of different combinations of treatment modalities as well as different sequences of RT or chemotherapy should be performed to learn more about their long-term effects.

## Conclusions

Though with limitations, this study indicated that the RT did not confer an additional cancer-specific or overall survival advantage beyond that achieved with surgery plus chemotherapy alone for LA NSCLC patients. However, for patients at T1/2 & N2/3, the chemotherapy-only group seems to be at a survival disadvantage compared with neoadjuvant or adjuvant RT groups. The findings of this study suggest that the benefit of additional neoadjuvant or adjuvant RT to chemotherapy may be linked to a proper selection of LA NSCLC patients who undergo surgery. The timing of radiotherapy should be decided on the premise of fully considering patients’ condition and the quality of life after treatment.

## Additional file

Additional file 1: **Table S1.** Baseline characteristics of the unmatched cohorts. **Table S2.** Multivariable Cox regression for unmatched dataset. (DOCX 20 kb)

## Abbreviations

AJCC: The American Joint Committee on Cancer
LA NSCLC: Locally advanced non-small cell lung cancer
RT: Radiotherapy
