# Supplementary material for: Long-term effects of neoadjuvant radiotherapy, adjuvant radiotherapy, and chemotherapy-only on survival of locally advanced non-small cell lung Cancer undergoing surgery: a propensity-matched analysis
Source: BMC Cancer. 2018 Nov 6;18:1067. doi: 10.1186/s12885-018-4900-x (PMC6219254; doi:10.1186/s12885-018-4900-x)
Supplement: Supplementary file 1 — Table S1. Baseline characteristics of the unmatched cohorts. Table S2. Multivariable Cox regression for unmatched dataset. (DOCX 20 kb) [file 12885_2018_4900_MOESM1_ESM.docx]

**Table S1.** Baseline characteristics of the unmatched neoadjuvant and adjuvant radiotherapy cohorts.

| Variable | Number of patients (%) | | | | | | | *p-*value |
| --- | --- | --- | --- | --- | --- | --- | --- | --- |
|  | Adjuvant RT  (N=599) | |  | Neoadjuvant RT  (N=479) | | Surgery + Chemotherapy-  Only (N=895) | |  |
| Age, years | 61.58±9.92 | |  | 60.16±9.66 | | 63.51±9.63 | | <.0001 |
| Sex |  |  |  |  |  |  |  | 0.0887 |
| Male | 309 | (51.59) |  | 276 | (57.62) | 466 | (52.07) |  |
| Female | 290 | (48.41) |  | 203 | (42.38) | 429 | (47.93) |  |
| Race |  |  |  |  |  |  |  | 0.0003 |
| White | 455 | (75.96) |  | 392 | (81.84) | 696 | (77.77) |  |
| Black | 59 | ( 9.85) |  | 60 | (12.53) | 98 | (10.95) |  |
| Other | 85 | (14.19) |  | 27 | ( 5.64) | 101 | (11.28) |  |
| Histology |  |  |  |  |  |  |  | 0.0118 |
| Adenocarcinoma | 289 | (48.25) |  | 185 | (38.62) | 415 | (46.37) |  |
| Squamous cell | 129 | (21.54) |  | 138 | (28.81) | 212 | (23.69) |  |
| Others | 181 | (30.22) |  | 156 | (32.57) | 268 | (29.94) |  |
| Grade |  |  |  |  |  |  |  | <.0001 |
| I/ II | 240 | (40.07) |  | 107 | (22.34) | 402 | (44.92) |  |
| III/ IV | 309 | (51.59) |  | 229 | (47.81) | 435 | (48.60) |  |
| Unknown | 50 | ( 8.35) |  | 143 | (29.85) | 58 | ( 6.48) |  |
| T classification |  |  |  |  |  |  |  | 0.0003 |
| T1 | 116 | (19.37) |  | 76 | (15.87) | 140 | (15.64) |  |
| T2 | 262 | (43.74) |  | 190 | (39.67) | 324 | (36.20) |  |
| T3 | 66 | (11.02) |  | 65 | (13.57) | 97 | (10.84) |  |
| T4 | 155 | (25.88) |  | 148 | (30.90) | 334 | (37.32) |  |
| N classification |  |  |  |  |  |  |  | <.0001 |
| N0/N1 | 123 | (20.53) |  | 94 | (19.62) | 329 | (36.76) |  |
| N2/N3 | 476 | (79.47) |  | 385 | (80.38) | 566 | (63.24) |  |
| Extent of Resection |  |  |  |  |  |  |  | 0.2142 |
| Lobectomy or Bilobectomy | 516 | (86.14) |  | 394 | (82.25) | 757 | (84.58) |  |
| Pneumectomy | 83 | (13.86) |  | 85 | (17.75) | 138 | (15.42) |  |

**Table S2**. Multivariable Cox regression for unmatched dataset.

| Variable | Cancer-Specific mortality | |  | All-cause mortality | |
| --- | --- | --- | --- | --- | --- |
|  | HR (95% CI) | *p-*value |  | HR (95% CI) | *p-*value |
| Age | 1.01(1.01, 1.02) | 0.0001 |  | 1.02(1.01, 1.02) | <.0001 |
| Sex |  |  |  |  |  |
| Male | *Ref.* |  |  | *Ref.* |  |
| Female | 0.83(0.73, 0.94) | 0.0048 |  | 0.81(0.71, 0.91) | 0.0006 |
| Race |  |  |  |  |  |
| White | *Ref.* |  |  | *Ref.* |  |
| Black | 0.93(0.75, 1.15) | 0.4808 |  | 0.92(0.76, 1.13) | 0.4394 |
| Others | 1.04(0.85, 1.27) | 0.7168 |  | 1.01(0.84, 1.22) | 0.9245 |
| Grade |  |  |  |  |  |
| I/ II | *Ref.* |  |  | *Ref.* |  |
| III/ IV | 1.20(1.04, 1.37) | 0.0099 |  | 1.22(1.07, 1.39) | 0.0021 |
| Unknown | 0.95(0.76, 1.18) | 0.6284 |  | 0.94(0.76, 1.16) | 0.5591 |
| Histology |  |  |  |  |  |
| Adenocarcinoma | *Ref.* |  |  | *Ref.* |  |
| Squamous cell | 0.84(0.71, 1.00) | 0.0563 |  | 0.89(0.76, 1.05) | 0.1658 |
| Others | 0.94(0.81, 1.09) | 0.4022 |  | 0.96(0.83, 1.10) | 0.5545 |
| T classification |  |  |  |  |  |
| T1 | *Ref.* |  |  | *Ref.* |  |
| T2 | 1.26(1.04, 1.52) | 0.0178 |  | 1.22(1.02, 1.45) | 0.0263 |
| T3 | 1.58(1.22, 2.06) | 0.0006 |  | 1.44(1.12, 1.84) | 0.0040 |
| T4 | 1.31(1.04, 1.66) | 0.0217 |  | 1.20(0.97, 1.50) | 0.0986 |
| N classification |  |  |  |  |  |
| N0 | *Ref.* |  |  | *Ref.* |  |
| N1 | 1.51(1.24, 1.85) | <.0001 |  | 1.37(1.14, 1.66) | 0.0010 |
| Extent of Surgery |  |  |  |  |  |
| Lobectomy or bilobectomy | *Ref.* |  |  | *Ref.* |  |
| Pneumectomy | 1.20(1.00, 1.43) | 0.0490 |  | 1.17(0.99, 1.38) | 0.0672 |
| Treatment regime |  |  |  |  |  |
| Neoadjuvant RT | *Ref.* |  |  | *Ref.* |  |
| Adjuvant RT | 1.07(0.89, 1.28) | 0.4771 |  | 1.11(0.94, 1.31) | 0.2145 |
| Surgery + Chemotherapy-Only | 1.06(0.89, 1.26) | 0.5169 |  | 1.09(0.93, 1.28) | 0.2943 |

PSM: propensity score matching, HR: Hazard Ratio.
